# Acculturation and use of health care services by Turkish and Moroccan migrants: a cross-sectional population-based study

**DOI:** 10.1186/1471-2458-9-332

**Published:** 2009-09-10

**Authors:** Thijs Fassaert, Arlette E Hesselink, Arnoud P Verhoeff

**Affiliations:** 1 Department of Epidemiology, Documentation and Health Promotion, Amsterdam Municipal Health Service, Amsterdam, the Netherlands; 2 Department of Social Medicine, Academic Medical Center/University of Amsterdam, Amsterdam, the Netherlands; 3 Department of Sociology and Anthropology, University of Amsterdam, Amsterdam, the Netherlands

## Abstract

**Background:**

There is insufficient empirical evidence which shows if and how there is an interrelation between acculturation and health care utilisation. The present study seeks to establish this evidence within first generation Turkish and Moroccan migrants, two of the largest migrant groups in present-day Western Europe.

**Methods:**

Data were derived from the Amsterdam Health Monitor 2004, and were complete for 358 Turkish and 288 Moroccan foreign-born migrants. Use of health services (general practitioner, outpatient specialist and health care for mental health problems) was measured by means of self-report. Acculturation was measured by a structured questionnaire grading (i) ethnic self-identification, (ii) social interaction with ethnic Dutch, (iii) communication in Dutch within one's private social network, (iv) emancipation, and (v) cultural orientation towards the public domain.

**Results:**

Acculturation was hardly associated with the use of general practitioner care. However, in case of higher adaptation to the host culture there was less uptake of outpatient specialist care among Turkish respondents (odds ratio [OR] = 0.90, 95% confidence interval [CI] = 0.82-0.99) and Moroccan male respondents (OR = 0.81, 95% CI = 0.71-0.93). Conversely, there was a higher uptake of mental health care among Turkish men (OR = 0.81, 95% CI = 0.71-0.93) and women (OR = 0.81, 95% CI = 0.71-0.93). Uptake of mental health care among Moroccan respondents again appeared lower (OR = 0.74, 95% CI = 0.55-0.99). Language ability appeared to play a central role in the uptake of health care.

**Conclusion:**

Some results were in accordance with the popular view that an increased participation in the host society is concomitant to an increased use of health services. However, there was heterogeneity across ethnic and gender groups, and across the domains of acculturation. Language ability appeared to play a central role. Further research needs to explore this heterogeneity into more detail. Also, other cultural and/or contextual aspects that influence the use of health services require further identification.

## Background

Generally, ethnic differences in use of health services are considered to be undesirable, but have been demonstrated repeatedly regarding a wide range of health problems [1-4]. Apart from the influence of socioeconomic status (SES) and health care need - i.e. ethnic minority status tends to correlate negatively with SES and health status [4,5] - reasons for ethnic disparities in access and use of health services are complex and often poorly understood [6]. Even when SES and health care need are taken into account, differences tend to persist, suggesting that other factors should be considered as well [4,7,8]. Identifying these factors for specific ethnic groups may help us to further understand the differences in health care use and so define strategies for preventive policy.

One factor that deserves closer examination is acculturation [9-13]. Acculturation has been associated with significant changes in health behaviour, health, and morbidity for ethnic minority groups [9,14-17]. Consequently, acculturation is considered to be an important variable in health psychology and behavioural medicine [9,14]. Usually, it is defined along two dimensions, expressing the degree of contact and participation in the larger society and maintenance of heritage culture and identity [18,19]. As such, the acculturation process is believed to have four outcomes, namely 'assimilation' (in which the old culture is rejected, and participation in the host culture is high), 'separation/traditionalism' (in which the old culture is preserved, and participation in the host culture is low), 'marginalization' (in which both cultures are rejected) and 'integration' (in which the original culture is preserved and participation in the host culture is high) [18,19]. Since most differences in health behaviour have been found between the traditional and assimilated conditions, many researchers have constructed one-dimensional acculturation scales, with traditionalism and assimilation as their extremes [14].

Several studies looking for the correlation between acculturation and services use suggest that increased participation in (or adaptation to) the host culture (i.e. integration and assimilation) is associated with higher service use [20-22]. However, the empirical evidence from Western European health care settings in support of this hypothesis is still rather poor. Differences compared to findings from the U.S. may be expected as a result of (i) variations between studies and countries with respect to the historical background of migration to the U.S. and the Netherlands (e.g. slavery, decolonisation, or labour migration), (ii) the definition of ethnic minority status (e.g. based on self-identification, religion, country of birth, or race), and (iii) health care systems (e.g. with or without general/family practitioners serving as gatekeepers to specialised mental health care, as well as compulsory health insurance in the Netherlands). Moreover, the empirical evidence that supports the notion of higher use of services in case of higher acculturation is in fact more heterogeneous than supposed, suggesting that the effect of acculturation may vary by ethnic and gender background [14,20,23-26].

Compared to the general Dutch population, differences in health status and use of health services by Turkish and Moroccan labour migrants - currently two of the most prominent ethnic minority populations in Western Europe - have been shown [4,8,27,28]. Although these differences tend to vary, depending on which migrant group or health service type is focused upon [29], the general impression is that both groups are well represented in general practice, whereas specialised health services, like outpatient care and mental health care, are underutilised in comparison to ethnic Dutch [4]. As far as we are aware, only one study explored the association between acculturation and health care use among Turkish and Moroccan migrants, showing that higher levels of adaptation and lower levels of cultural traditionalism increased the use of mental health services [9]. The present study seeks to establish if and how acculturation is associated with use of general practitioner, outpatient specialist and mental health care, among first-generation (i.e. foreign-born) Turkish and Moroccan migrants in the Netherlands, while taking into account predisposing and need factors that may confound the association between acculturation and use of health services [30].

## Methods

### Study population

Participants were recruited as part of a cross-sectional population-based health survey (the Amsterdam Health Monitor, or AHM) [31-35]. The survey was carried out in 2004 by the Amsterdam Municipal Health Service (GGD) in collaboration with the National Institute for Public Health and the Environment (RIVM). Amsterdam consists of thirteen districts and the sample for our study was drawn from five of them. These five contain a population that was representative concerning socioeconomic status and ethnicity for the total population of Amsterdam. The sample was stratified by ethnic background and five age groups (18-34, 35-44, 45-54, 55-64 and 65 years or older). Respondents were invited for an interview and medical examination in a community health centre. All interviews were conducted in the language of choice of the respondent (i.e. Dutch, Turkish, Moroccan-Arabic or Berber). The overall response rate among ethnic Dutch, Turkish and Moroccan subjects was 45%. Regarding ethnic background, response was lowest among Moroccans (38.8%) and ethnic Dutch (45.9%) compared with Turkish (49.6%; p < 0.001). Among the Turkish and Moroccan group there were significantly more women (p < 0.001) and response was also lowest in the youngest (18-34 years) age group. After weighting the data for age, gender en ethnicity, respondents appeared to have an annual income and unemployment rate that was comparable to that of the total population of Amsterdam in 2004 [34]. That is, in 2005 around 6.7% of the working class was unemployed and thirty one percent of the Amsterdam citizen in 2004 had an annual income of less than €15.800, in 54% this was between €15.800 and €39.900, and 15% had an annual income of at least €39.900. These percentages do correspond with the percentages found in our total study population. These data were not available for ethnic groups separately. All respondents in the AHM signed an informed consent form. All study procedures for the AHM were checked and approved by the Medical Ethical commission of the Amsterdam Academic Medical Center.

### Acculturation

The central variable in this study was acculturation. Firstly, ethnic self indication indicated acculturation. Accordingly, subjects had to point out to which ethnic group they felt they belonged: (i) ethnic Moroccan or Turkish (ii), both ethnic Dutch and Moroccan or Turkish, or (iii) ethnic Dutch. Secondly, acculturation was measured by a structured questionnaire. This questionnaire was originally designed by Martens [36] and further developed and applied in studies by Nierkens et al. [37], Hosper et al. [16,38], and Dijkshoorn et al. [27]. The instrument is based on Berry's two-dimensional model, which discriminates between orientation towards the majority culture versus culture of origin, and social contacts with the host population versus contacts with people from the culture of origin. As such, the questionnaire measures the following domains: (a) emancipation (6 items, e.g. 'Education is more important for boys than for girls' or 'It is better that men take responsibility over financial issues'; range 6-18); (b) social interaction with ethnic Dutch (3 items, e.g. 'Do you socialise with ethnic Dutch?' or 'How many of your best friends are ethnic Dutch'; range 3-9); (c) communication in Dutch within one's private domain (3 items, e.g. 'Do you speak Dutch with your children?'; range 3-9); and (d) cultural orientation towards the public domain (5 items, e.g. 'About how often do you watch Dutch movies, television or video programmes?' and 'Do you buy your groceries mostly in a Turkish/Moroccan shop, a Dutch supermarket, or both?'; range 5-15). To validate the domain structure of the questionnaire in our sample, we conducted a Principal Component Analysis (PCA) with Varimax rotation and a forced four-factor solution. These four factors had eigenvalues of 3.28 (cultural orientation), 2.21 (emancipation), 1.50 (social interaction) and 1.24 (communication in Dutch). With the exception of two items, all items had factor loadings of 0.40 or higher on the relevant factors. Furthermore, the Cronbach's alpha's for the different domains were 0.67 (emancipation), 0.65 (social interaction), 0.63 (communication), and 0.62 (public domain). Sum scores on these acculturation domains were calculated as to indicate a higher degree of orientation towards Dutch culture.

### Other measurements

The outcome measure for this study was health services utilisation, measured by way of self-report. A distinction was made between visits to a general practitioner (GP) (yes or no), outpatient specialist (yes or no) or any health care visits for mental health problems (yes or no). Use of services referred to the last two months that preceded the interview.

Similar to Uiters et al. [8], health care need was estimated with the first item of the SF-12 questionnaire [39] (i.e. 'In general, would you describe your health as: (i) excellent, (ii) very good, (iii) good, (iv) poor, or (v) very poor') and the number of chronic illnesses. The latter was measured by asking respondents to indicate whether they had suffered from a selection of chronic diseases in the past 12 months, and if they visited a GP for that disease. The selection of chronic illnesses was derived from the Permanent Survey on Living Conditions (POLS) and included, amongst others, cancer, diabetes, hypertension, and arthritis [40]. There were 11 illnesses, and thus the total scores of the number of chronic conditions ranged between 0 and 11.

Predisposing factors included demographic information (i.e. sex, age and ethnicity) and socioeconomic status (SES). Ethnicity was classified according to the definition of Statistics Netherlands, which is based on (self-reported) country of birth of the respondent and his/her parents. First-generation migrants are defined as subjects who were born in Turkey or Morocco, and of whom at least one parent was born in Turkey or Morocco as well [41]. Second generation migrants were born in the Netherlands, but at least one of both parents was born in Turkey or Morocco. Since only 41 second generation Turkish and Moroccan migrants participated in the AHM, this study focuses on first-generation migrants only. SES was indicated by the level of education, and divided into two categories: primary school or lower, and secondary/vocational school, high school or higher. This distinction was made because the educational level in the present sample was generally (very) low (more than 65% of the Turkish and Moroccan respondents were classified as low).

### Statistical Analyses

All analyses were conducted for the total study population as well as for Moroccan and Turkish men and women separately, taking into account that Moroccan and Turkish migrants have somewhat different ethnocultural backgrounds, that there are gender specific patterns in health status and health care utilisation, and that gender may interact with both acculturation and health [23-26]. Only participants with complete information were included in the analyses. Logistic regression analysis was carried out. Interaction terms were included to study whether the association between acculturation and health services use differed between Moroccan and Turkish respondents, and between gender groups. In case of statistically significant interaction, stratified analyses were presented. All statistical analyses were done in SPSS 15.0 for Windows [42].

## Results

Of 770 first generation Turkish and Moroccan respondents in the AHM, the majority (83.9%) had complete information on all relevant variables, leading to a sample size of 646 subjects for this study (table 1). There were no statistically significant differences between respondents with complete information and those without complete information regarding ethnic background, gender, age, education, general health, the number of chronic health conditions, GP-care, outpatient care, mental health care, ethnic self-identification, or any of the acculturation subscales. Table 1 presents participant information for the total sample, and for Turkish and Moroccan men and women separately. There were statistically significant differences between Turkish and Moroccan men and women regarding age, educational level, self-reported health and chronic conditions, and uptake of mental health services. Concerning acculturation, differences were found with respect to social interaction with ethnic Dutch and cultural orientation. No significant differences were found for communication and emancipation. Finally, table 1 shows that there was insufficient variance with respect to the variable of ethnic self-identification for this variable to have predictive value. As a result this variable was not introduced in the regression analyses.

**Table 1:** Sociodemographic characteristics of the study population, per ethnic group (N = 646)

	**All**	**Turkish**		**Moroccan**		
	**(N = 646)**	**Men** **(N = 170)**	**Women** **(N = 188)**	**Men** **(N = 157)**	**Women** **(N = 131)**	**p-value**
**Ethnicity**						
Turkish (%)	55.4	-	-	-	-	
Moroccan (%)	44.6	-	-	-	-	
**Sex**						
Female (%)	49.4	-	-	-	-	
**Age**	48.6 (13.0)	49.7 (12.0)	44.7 (12.9)	53.3 (12.8)	46.9 (12.9)	< 0.001
**Education**						
more than primary (%)	34.5	42.9	33.5	32.5	27.5	0.035
**Self reported health**						
excellent (%)	3.7	5.3	3.2	3.8	2.3	0.037
very good (%)	5.3	9.4	3.2	3.2	5.3	
good (%)	35.6	35.9	30.9	40.1	36.6	
moderate (%)	39.8	31.8	42.0	41.4	45.0	
bad (%)	15.6	17.6	20.7	11.5	10.7	
**N chronic conditions** (range = 0-11; mean(sd.))	1.8 (1.7)	1.5 (1.6)	2.2 (1.8)	1.6 (1.6)	1.7 (1.6)	< 0.001
**Use of health service**						
general practitioner (%)	60.8	55.3	62.2	61.8	64.9	0.347
outpatient specialist (%)	25.2	25.3	23.9	23.6	29.0	0.709
mental health care (%)	10.2	15.3	13.3	6.4	3.8	0.002
**Ethnic self-identification**						
Dutch/bi-ethnic (%)	8.8	9.4	8.0	10.8	6.9	0.650
Turkish or Moroccan (%)	91.2	90.6	92.0	89.2	93.1	
**Cultural orientation** (range = 5-15; mean(sd.))^1^	10.0 (2.6)	10.0 (2.3)	9.5 (2.6)	10.5 (2.5)	10.0 (2.8)	0.004
**Emancipation** (range = 6-18; mean(sd.))^1^	15.0 (2.8)	14.8 (2.9)	15.3 (2.5)	14.8 (3.0)	15.1 (2.8)	0.253
**Communication in Dutch** (range = 3-9; mean(sd.))^1^	6.0 (2.9)	6.3 (3.0)	5.9 (2.9)	5.9 (2.7)	5.8 (2.9)	0.419
**Social contacts with** **ethnic Dutch** (range = 3-9; mean(sd.))^1^	4.3 (1.6)	4.7 (1.8)	4.2 (1.5)	4.4 (1.7)	4.1 (1.6)	0.004

^1 ^a higher score indicates higher acculturation

Table 2 shows the first results from the regression analyses. With respect to GP care there was no interaction between acculturation and ethnic background, nor between acculturation and gender. Therefore stratified analyses were not conducted. The table shows that there was no relation between acculturation and GP care. Instead the main correlates of GP care were worse self reported health and higher number of chronic conditions. In addition, higher age was also related to higher GP care uptake.

**Table 2:** Association between acculturation and use of general practice care (odds ratios and 95% confidence intervals)§

	**Total**
	**(N = 646)**
Moroccan ethnicity^1^	1.18 (0.82-1.70)
Sex^2^	0.77 (0.53-1.12)
Age ^3^	1.03 (1.01-1.05)*
Education ^4^	1.23 (0.79-1.92)
Self reported health ^5^	1.90 (1.51-2.40)**
Number of chronic conditions ^6^	1.26 (1.09-1.45)*
Cultural orientation ^7^	1.04 (0.92-1.19)
Emancipation ^7^	1.00 (0.94-1.07)
Communication in Dutch ^7^	0.95 (0.89-1.02)
Social interaction ^7^	1.08 (0.99-1.19)

§ There was no interaction between acculturation and ethnicity and/or gender; analyses were not presented for subgroups.

* p < 0.05 ** p < 0.001

^1 ^'Turkish ethnicity' served as reference category

^2 ^'Male gender' served as reference category

^3 ^Continuous variable (18 years or older). Each step equals +1 year

^4 ^'Primary school at most' served as reference category

^5 ^'Excellent self reported health' served as reference category

^6 ^Continuous variable (range 0-11). Each step equals +1 chronic condition

^7^A higher score indicates higher acculturation

There was statistically significant interaction between acculturation (communication) and ethnicity with respect to specialist care (p = 0.032; table 3). The analyses were therefore stratified according to ethnicity. In the Turkish group higher acculturation regarding communication was associated with less uptake of specialist care. Among Moroccans there was no such relation. In the Moroccan sample there was also interaction between acculturation and gender (p = 0.044); a higher degree of emancipation was associated with less uptake of specialist care among men, but among Moroccans women there was no such association.

**Table 3:** Association between acculturation and use of outpatient specialist care (odds ratios and 95% confidence intervals)§

	**Turkish**	**Moroccan**	
	**(N = 358)**	**Men (N = 157)**	**Women (N = 131)**
Moroccan ethnicity^1^	---	---	---
Sex^2^	1.28 (0.74-2.24)	---	---
Age ^3^	1.01 (0.98-1.03)	1.03 (0.99-1.08)	1.00 (0.95-1.04)
Education ^4^	1.15 (0.60-2.18)	1.71 (0.55-5.26)	0.27 (0.08-0.95)*
Self reported health ^5^	1.73 (1.23-2.42)*	0.85 (0.48-1.51)	1.19 (0.65-2.17)
Number of chronic conditions ^6^	1.17 (0.98-1.40)	1.41 (1.05-1.89)*	1.33 (0.97-1.82)
Cultural orientation ^7^	1.14 (0.95-1.36)	0.76 (0.54-1.07)	1.17 (0.84-1.63)
Emancipation ^7^	1.04 (0.94-1.14)	0.81 (0.71-0.93)*	1.01 (0.86-1.18)
Communication in Dutch ^7^	0.90 (0.82-0.99)*	1.04 (0.87-1.24)	1.07 (0.92-1.24)
Social interaction ^7^	1.04 (0.91-1.18)	0.93 (0.75-1.16)	1.23 (0.90-1.54)

§ There was interaction between acculturation and ethnicity, and within the Moroccan subpopulation there was also interaction between acculturation and gender.

* p < 0.05 ** p < 0.001

^1 ^'Turkish ethnicity' served as reference category

^2 ^'Male gender' served as reference category

^3 ^Continuous variable (18 years or older). Each step equals +1 year

^4 ^'Primary school at most' served as reference category

^5 ^'Excellent self reported health' served as reference category

^6 ^Continuous variable (range 0-11). Each step equals +1 chronic condition

^7^A higher score indicates higher acculturation

With respect to mental health care there was also statistically significant interaction between the communication subscale and ethnic background (p = 0.033; table 4). Among Turkish there was additional interaction between communication and gender (p = 0.003) and between social interaction and gender (p = 0.02). As a result, stratified analyses were done based on ethnic background and gender, the latter only among Turkish. Subgroup analyses showed that higher acculturation regarding communication was associated with higher uptake of mental health services among Turkish men, while the opposite appeared to be the case for Moroccan subjects. Furthermore, higher social interaction was related to higher uptake of mental health services among Turkish women.

**Table 4:** Association between acculturation and use of mental health care (odds ratios and 95% confidence intervals)§

	**Turkish**		**Moroccan**
	**Men (N = 170)**	**Women (N = 188)**	**(N = 288)**
Moroccan ethnicity^1^	---	---	---
Sex^2^	---	---	2.67 (0.75-9.52)
Age ^3^	0.95 (0.90-0.99)*	1.00 (0.95-1.04)	0.91 (0.85-0.97)*
Education ^4^	0.62 (0.20-1.88)	0.54 (0.16-1.84)	1.51 (0.36-6.28)
Self reported health ^5^	2.15 (1.15-4.04)*	1.84 (0.94-3.58)	3.15 (1.14-8.66)*
Number of chronic conditions ^6^	1.27 (0.92-1.76)	1.19 (0.88-1.60)	1.61 (1.03-2.54)*
Cultural orientation ^7^	0.77 (0.56-1.05)	1.31 (0.92-1.86)	1.32 (0.86-2.02)
Emancipation ^7^	0.93 (0.80-1.09)	1.00 (0.82-1.21)	0.98 (0.78-1.24)
Communication in Dutch ^7^	1.26 (1.06-1.49)*	0.83 (0.69-1.01)	0.74 (0.55-0.99)*
Social interaction ^7^	0.96 (0.76-1.22)	1.27 (1.01-1.61)*	1.07 (0.80-1.45)

§ There was interaction between acculturation and ethnicity, and within the Turkish subpopulation there was also interaction between acculturation and gender.

* p < 0.05 ** p < 0.001

^1 ^'Turkish ethnicity' served as reference category

^2 ^'Male gender' served as reference category

^3 ^Continuous variable (18 years or older). Each step equals +1 year

^4 ^'Primary school at most' served as reference category

^5 ^'Excellent self reported health' served as reference category

^6 ^Continuous variable (range 0-11). Each step equals +1 chronic condition

^7^A higher score indicates higher acculturation

## Discussion

In this study we focused on the association between acculturation and the use of health services within a population-based sample of first generation Turkish and Moroccan migrants in the Netherlands. Generally, there was only a moderate association between acculturation and health care utilisation, in that predominantly the domain communication in Dutch was related to utilisation, and GP care was not related to acculturation at all. Instead, health care utilisation was strongly related to subjective and objective measures of health care need, namely self-reported health status and the number of chronic conditions. If need factors, rather than factors like ethnic background or - as in this case - acculturation, are major determinants of health care utilisation, this is essentially a positive observation, since this can be argued to be an indicator of equity in health care access [4].

Some positive associations between acculturation and use of health care services were found. Among the Turkish group acculturation was generally associated with the use of mental health care services; more communication in Dutch (within one's private domain) was associated with more use of mental health care amongst Turkish men, while among women this was the case for social interaction. The direction of these associations is in line with the common hypothesis that increasing participation in the host culture is associated with higher service use. It is also in line with findings among other migrant groups in the U.S. [20], and - more importantly - with recent findings from a study in the Netherlands focussing on the use of mental health services [9].

However, among Moroccan subjects the association ran opposite while more emancipation was associated with less uptake of outpatient care (males) and more communication in Dutch was associated with less mental health care (males and females). A possible explanation for the association between more communication in Dutch and less uptake of mental health care among Moroccans may be a result of the methodology used in our study. That is, although general health status was accounted for, a specific measure of mental health was lacking. It could be that Moroccans who were better able to communicate in Dutch experienced less psychological stress and consequently a lower mental health care need. For example, in a previous study we found that lacking the skills to live/participate in the Dutch society largely related to mastery of the Dutch language, was associated with more psychological distress [43]. If this is the case indeed, then the question of course rises why we found a comparable association with outpatient specialist care and the opposite trend among Turkish respondents.

Another explanation for the aforementioned reverse relationship among Moroccans regarding mental health care utilisation may be found in the absence or availability of social support. That is, compared to Turkish migrants, first-generation Moroccans in the Netherlands tend to have smaller social networks, which often do not extend beyond their direct families [44]. Possibly, Moroccans with better Dutch language skills are less likely to become socially isolated, may be more likely to have alternative sources of (informal) support in case of health care need, and are consequently less likely to apply for mental health care.

Finally, an explanation of this adverse relationship, especially regarding health care from outpatient specialists, may be that higher levels of adaptation concur with a better knowledge of the Dutch health care system and the role of Dutch GPs. GPs in the Netherlands act as gatekeepers to outpatient specialist care, while in Turkey for example it is common practice to visit medical specialists directly. It might be that those who displayed better skills for living in Dutch society were more aware of this than those who did not, and were therefore more likely to remain in care in general practice. One argument against this explanation, however, lies in the observation that we found no association between acculturation and uptake of care in general practice.

The latter observation is important, because in our view it indicates and supports the notion that GP care in the Netherlands has a low threshold and is highly accessible. Recently, Uiters et al. already concluded that the gate keeping role of general practitioners in the Netherlands functions equally effectively among ethnic minority groups compared to the ethnic Dutch population [8]. The observation that acculturation was associated with outpatient specialist and mental health care utilisation seems to suggest that if services are less accessible, or when culturally defined stigma and taboo come into play (as is the case for mental health problems), acculturation becomes more relevant as a concept in health services research.

This study has limitations. First, due to its cross-sectional design, no conclusions are allowed on the directionality of the results. For example, it is conceivable that ill health (including mental health), indicated by higher use of services, limits the ability of respondents to acculturate. Moreover, ill health may have resulted in a disproportionately negative self-evaluation regarding one's own acculturation skills. A second limitation concerns the generalisability of our results, which may have been compromised by the relatively high non-response. Selective non-response might have occurred, as people who do not use health care services may also be less willing to participate in research. However, the age/sex distribution in the responding sample was reasonable. The non-response also caused a relatively low number of respondents in each analysis. Considering the moderate range of most of the statistical associations observed, one may wonder if the associations are clinically significant. Some of the confidence intervals were extremely wide. Another source of bias might include the fact that measures were self-reported, although self-report measures have been found to be reasonably valid estimators for comparisons between migrant groups in the Netherlands [45].

Additionally, the acculturation instrument was strongly focused on measuring adaptation, or assimilation. As such, no statements can be made about the role of the second dimension of Berry's model of acculturation (i.e. maintenance of heritage culture and identity), while in previous studies it has been shown that this is also a very important aspect. On the other hand, measures of acculturation traditionally focus on language ability alone. Indeed, it can be derived from the results that, although the associations between acculturation and health care utilisation were limited and heterogeneous, communication in Dutch was one of the most important and central aspects of acculturation. However, other dimensions played a role of significane as well. The finding that increasing social interaction by Turkish women was associated with increasing mental health care, for example, is noteworthy in the light of marginalisation of Muslim migrant groups in Western countries [46]. That is, if we consider increasing mental health care utilisation by Turkish women a good development, than current political and social developments, by some labelled as "Islamophobia" can be considered a threat in this respect.

Although the factor structure of the acculturation was supported by our data, reliability of the subscales was not strong. According to Nunnaly, Cronbach's alpha coefficients should be 0.70 or higher [47]. However, lower values - such as in our study - have also been presented as adequate: by citing other studies [48,49], Milfont and Gouveia [50] argue why reliabilities in the range 0.60 and 0.70 can be regarded as adequate as well, and that if samples are sizes larger than 100 (which is the case for all subgroups in our analyses) alpha coefficients greater than 0.40 are acceptable. Furthermore, the Cronbach's alpha we found corresponded with those found in another Dutch study carried out by Hosper et al. [16] using the same instrument (Cronbach's alpha of 0.64 is presented for the combined scale and a Cronbach's alpha of 0.84 and 0.80 on the social interaction scale).

Finally, and unfortunately, the acculturation scale was applied only in the ethnic minority groups and not among ethnic Dutch. Comparisons between ethnic Dutch, Moroccan and Turkish respondents were thus not feasible. Adjusting and applying acculturation measures for use among original inhabitants of host countries is not common use in health services research, but is a good suggestion for further studies. For example, it would have been very interesting if we could have seen how the ethnic Dutch scored on the emancipation subscale.

## Conclusion

The results of our study concur to some degree with the general idea that increasing participation in (or adaptation to) a host society (i.e. integration and assimilation) is related to more health services utilisation. Specific associations varied according to service type, ethnic background and gender group. An implication for clinical practice follows from the central role language ability played, considering that the domains of communication and social interaction are closely related to language fluency. Resulting from that, language support in clinical practice is and remains a major issue. Naturally, focusing on improving mastery of the Dutch language is not always feasible for first-generation migrants, for example as a consequence of illiteracy. Increasing efforts to assist and educate migrant patients in (mental) health care in their own languages are however a good alternative. With respect to future research, it should be mentioned that we only studied the use of care and not the quality of care which is generally suggested to be worse when minorities have a lower language proficiency. Future research might therefore be directed at the relation between acculturation and the quality of care.

## Competing interests

The authors declare that they have no competing interests.

## Authors' contributions

TF and AEH formulated the research question, performed the statistical analyses and drafted the manuscript. APV contributed to the manuscript with his interpretation of results and comments on earlier drafts. All authors read and approved the final version of the manuscript.

## Pre-publication history

The pre-publication history for this paper can be accessed here:


